# Stimulating the parietal cortex by transcranial direct current stimulation (tDCS): no effects on attention and memory

**DOI:** 10.3934/Neuroscience.2021002

**Published:** 2020-11-18

**Authors:** Mirela Dubravac, Beat Meier

**Affiliations:** Institute of Psychology, University of Bern, Bern, Switzerland

**Keywords:** brain stimulation, tDCS, parietal cortex, attention, memory, neuronal networks

## Abstract

Selective attention is relevant for goal directed behavior as it allows people to attend to task-relevant target stimuli and to ignore task-irrelevant distractors. Attentional focus at encoding affects subsequent memory for target and distractor stimuli. Remembering selectively more targets than distractors represents memory selectivity. Brain imaging studies suggest that the superior parietal cortex is associated with the dorsal attentional network supporting top-down control of selective attention while the inferior parietal cortex is associated with the ventral attentional network supporting bottom-up attentional orienting. To investigate the roles of the dorsal and ventral networks in the effect of selective attention during encoding on long-term memory, we stimulated the left superior and the right inferior parietal cortex. Building on previous work, we applied transcranial direct current stimulation (tDCS) during a study phase where pictures and words were presented simultaneously and participants had to switch between a picture and a word decision. A subsequent recognition test assessed memory for target and distractor pictures and words. We hypothesized that a relative increase in activity in the dorsal network would boost selective attention while increased activity in the ventral network would impair selective attention. We also expected to find corresponding effects on memory. Enhanced selective attention should lead to higher memory selectivity, while impaired selective attention should lead to lower memory selectivity. Our results replicated that task switching reduced memory selectivity. However, we found no significant effects of tDCS. Thus, the present study questions the effectiveness of the present tDCS protocol for modulating attention during task switching and subsequent memory.

## 1. Introduction

In order to navigate successfully through our environment (e.g., driving a car), we rely on two attentional systems; top-down focusing (e.g., on the road) and bottom-up orienting (e.g., to incoming cyclists). The interaction between the two systems allows us goal oriented behavior while flexibly adapting to changing environments [1],[2]. However, attention control is costly as it consumes cognitive resources needed for solving cognitively demanding tasks. For example, switching between two tasks leads to switch costs not only for immediate performance but also for subsequent memory of task-relevant targets [3]–[7]. For task-irrelevant distractors, however, a memory benefit occurs [5],[6],[8]. On switch trials, when the appropriate task-set is reconfigured, attention is broadened so that more distractors are encoded at the expense of targets. This explanation is in line with fMRI studies suggesting a correspondence between attention control and episodic retrieval in the posterior parietal cortex [9]. As episodic memory can be modulated by stimulating parietal substrates of attention during encoding [10], in the present study, we applied transcranial direct current stimulation (tDCS) over the parietal cortex to establish a causal link between the activity of two neural networks involved in top-down and bottom-up control during task switching. Based on previous behavioral as well as brain imaging and stimulation findings, we assumed a corresponding effect on subsequent memory selectivity.

Attention is not a unitary construct and is neither associated with a single circumscribed brain area. Rather, attention is a result of the interaction of different brain areas that are organized in networks. Specifically, the dorsal attentional network, which includes the superior parietal and frontal cortex, is involved in top-down selection of goal-relevant stimuli, while the ventral frontoparietal network is involved in bottom-up selection of salient stimuli [1],[11]. That is, the ventral system interrupts the dorsal system to direct attention towards potentially relevant stimuli (e.g., fast moving objects or animals signaling danger). This dual-attention perspective is supported by fMRI studies showing a relationship between parietal cortex activity during encoding and subsequent memory performance [9]. Increased activity in the dorsal parietal cortex is associated with subsequent memory success while increased activation in the ventral parietal cortex is associated with subsequent memory failure, suggesting that hippocampal encoding mechanisms are sensitive to attention modulations [9],[12]–[14].

An elegant demonstration of the relationship between selective attention and subsequent memory selectivity comes from studies that used a task switching procedure as an incidental study phase and assessed subsequent memory for previously presented items [3]–[7]. In these studies, participants were asked to classify stimuli (i.e., pictures and words) according to either one of two classification tasks signaled by a cue. Switching tasks is typically associated with more errors and longer reaction times compared to repeating a task suggesting more efficient attention control on repeat trials [15]. As a consequence, task-relevant target stimuli are better remembered if they appeared on a repeat trial, while task-irrelevant distractor stimuli are better remembered if they appeared on a switch trial [5],[6]. That is, task switching impairs selective attention and selective memory. Most relevant for the present study, event related potentials around stimulus presentation and functional brain activity point to the parietal cortex as a key region for task switching and subsequent memory effects [7],[16],[17]. However, the roles of dorsal and ventral parts of the parietal cortex for memory are not well understood.

Brain stimulation techniques proved useful in establishing a causal link between brain activation and behavior. As a safe noninvasive method to experimentally manipulate neuronal activity in certain brain areas, tDCS has been applied over frontal and parietal cortex areas to modulate cognition. For example, tDCS over the right intraparietal sulcus affected the detection of target and distractor stimuli, suggesting an involvement of the dorsal network in top-down control of selective attention [18]. More relevant for the present purpose is a study that applied tDCS over the superior and inferior parietal lobes targeting the dorsal and ventral attentional networks [10]. Participants were asked to learn a word list while receiving tDCS stimulation over the parietal cortex. They were assigned to one of two stimulation conditions. The first condition represented the selective attention condition entailing anodal stimulation over the left superior parietal cortex (a substrate of selective attention) and cathodal stimulation over the right inferior parietal cortex (a substrate of orienting). The second condition represented the orienting attention condition entailing the opposite polarity of stimulation. After the encoding phase, a recognition memory test was administered without tDCS. Participants in the selective attention condition recognized more words than participants in the orienting attention condition, suggesting that oppositional tDCS of parietal parts of two antagonistic attention networks modulates episodic encoding.

Following up on this study, we assume that the selective attention network is responsible for the benefit in memory performance, and thus we should find a dissociation between the two networks for memory selectivity. In the present study, we combined Richter and Yeung's [5],[6] paradigm with Jacobson et al.'s [10] tDCS protocol. Participants completed a task switching procedure while active or sham tDCS was applied over the parietal cortex. In the stimulation conditions, the exact same protocols were used as in Jacobson et al.'s study [10]. Oppositional tDCS was applied over the left superior and the right inferior parietal cortex. Anodal stimulation enhances neuronal excitability by depolarizing the membrane potentials of the underlying neurons. Conversely, cathodal stimulation reduces neuronal excitability by hyperpolarizing the membrane potentials of the underlying neurons. We reasoned that anodal stimulation of the superior parietal cortex would enhance activity in the dorsal network and that cathodal stimulation of the inferior parietal cortex would reduce activity in the ventral network. Together, this stimulation setup should enhance selective attention while the opposite polarity should enhance orienting. To establish a baseline and for better comparability with previous studies, we also included a sham control condition. The following recognition test consisted of pictures and words that were presented on either repeat or switch trials and that were either attended or unattended during task switching.

Based on Richter and Yeung's studies [5],[6], we expected switch costs on immediate performance and subsequent memory selectivity. Based on the dual attention theory and the study by Jacobson et al. [10], we predicted higher memory selectivity for participants in the selective attention condition compared to the baseline and even lower memory selectivity for the orienting attention condition. Critically, an interaction between task switching and stimulation condition would indicate that the effect of task switching on memory selectivity relies on the relative activity levels of the two attentional networks and would support the view that task switching reduces memory selectivity by impairing selective attention during encoding. To anticipate the main results, we replicated the switch costs on immediate performance and memory selectivity, but did not find any significant effects of tDCS.

## 2. Materials and methods

### 2.1. Participants

Sixty right handed participants (34 females and 26 males) aged between 18 and 28 years (*M* = 22, *SD* = 2) took part in the study. They were randomly assigned to one of three stimulation conditions (dorsal-anodal, sham, ventral-anodal). The investigator supervised the whole procedure while undergraduate students tested and interacted with the participants. Both, students and participants were blind with respect to stimulation condition. All participants gave written consent. The local ethics committee of the University of Bern approved the study.

### 2.2. Design

The experiment was a 3 × 2 × 2 mixed design. It consisted of the between-subjects factor stimulation condition (dorsal-anodal, sham, ventral-anodal) and two within-subject factors; attention (target vs. distractor), and transition type (repeat vs. switch trial). In each condition were 20 participants.

### 2.3. tDCS

**Figure 1.**
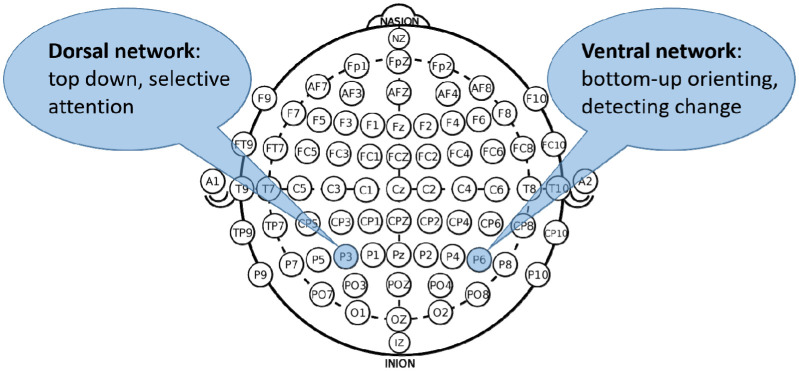
Schematic depiction of electrode positions.

tDCS was based on the protocol of Jacobson et al. [10]. Saline soaked sponge electrodes sized 5 × 5 cm and a DC Brain Stimulator Plus device (neuroConn, Ilmenau, Germany) were used. For stimulation of the left superior parietal cortex the electrode was placed over P3 and for stimulation of the right inferior parietal cortex the electrode was placed over P6. In the dorsal-anodal condition the anode was placed over P3 and the cathode over P6. In the ventral-anodal condition the cathode was placed over P3 and the anode over P6. In the sham condition the electrodes were placed as in the active stimulation conditions but the current was turned off after 30 s. Figure 1 shows a schematic presentation of the electrode positions. Stimulation was set at 1 mA. Fade in and fade out was set to 30 s each. The duration of tDCS depended on the time participants needed to complete the tasks (approx 10 min filler tasks for wash-in and 10 min task switching). After completing the critical task switching phase, tDCS was turned off. The total stimulation duration was approximately 20 min.

### 2.4. Stimuli

We adopted the stimuli from Richter and Yeung [5]. The set consisted of 288 words and 288 pictures. The words (originally from Poldrack et al., 1999 [19]) were abstract and concrete nouns translated into German and one to four syllables long. The pictures were monochrome photographs of natural and man-made objects on a black background (Hemera Photo Objects, Hull, Quebec, Canada). Words were printed in brown Arial font and superimposed over the pictures. Pictures and words were paired pseudo randomly to ensure an equal number of the four category combinations (abstract noun + man-made object, abstract noun + natural object, concrete noun + man-made object, concrete noun + natural object). The picture-word associations were held constant. The pairs were counterbalanced across participants.

### 2.5. Procedure

Participants were tested individually. The electrodes were placed over P3 and P6 and the stimulation was started (and turned off after 30 s in the sham condition). First, participants filled out the Edinburgh handedness inventory [20] and completed an unrelated filler task, assuring that at least 10 minutes elapsed between the start of the stimulation and the start of the experimental trials of the task switching procedure. After task switching the stimulation was turned off (in the case of the active stimulation conditions) and the electrodes were removed. Participants performed several filler tasks during a 20 min retention interval [10]. This extended retention interval served to make sure that the stimulation would not carry over to the recognition test. As tDCS may induce pain sensations [21] and to ensure that potential tDCS effects were not due to different levels of pain, we administered a numeric rating scale for pain. This scale was adapted from Hawker et al. [22]. Further, we asked participants to rate the (un-) pleasantness of their sensations on a 7-point scale. After completion, participants were debriefed, thanked and dismissed. In the following we describe the critical task switching procedure and the recognition test.

#### 2.5.1. Task switching

**Figure 2.**
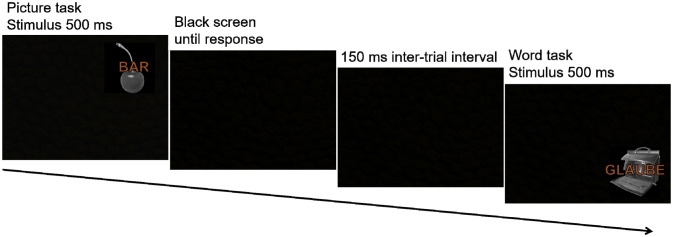
Two example trials of the task switching procedure. On the first trial the picture is the target and the word is the distractor as the picture task demands attention to be directed towards the picture. On the second example trial the picture is the distractor and the word the target.

Participants were instructed to categorize pictures as man-made or natural objects and words as abstract or concrete nouns as fast and correctly as possible. Participants gave their responses by keypress with their left middle and index fingers for the word task (x-key for abstract and c-key for concrete nouns) and the right middle and index fingers for the picture task (n-key for natural and m-key for man-made objects). The position of the picture-word pair on the screen cued the task [3],[23]. If the pair appeared in the upper half of the screen, participants had to solve the picture task and if it appeared in the lower half, they had to solve the word task. Participants were informed that the stimuli would appear successively in adjacent quadrants, in continuous, clockwise rotation: top left, top right, bottom right, bottom left, top left, and so on. The stimuli were presented for 500 ms followed by a blank screen until participant's response. The next trial started after 150 ms. Figure 2 depicts the trial procedure. Participants practiced the task for 20 trials. The practice block repeated until the participant reached a minimum of 80% correct answers. After ensuring participant's comprehension of the task, the experimental block started with four warm-up trials that were discarded from analysis. The experimental block consisted of 192 experimental trials. In total, the task lasted for approximately 10 minutes.

#### 2.5.2. Recognition test

Participants were informed that they will see pictures and words again, and that some of them were already presented in the previous task. They were instructed to identify these items by pressing the b-key for old and the n-key for new items in a forced-choice recognition test. The stimulus was presented in the middle of the screen until a key was pressed. After every “old” response a remember/know judgement was requested [3],[24],[25]. Participants had to press “1” if they were sure they remembered the item (recollection) and “2” if they had a feeling of knowing (familiarity). Words and pictures were tested in separate blocks. Two short practice blocks with four trials each, were administered before the experimental blocks. To attenuate the picture-superiority-effect [26], the word block was always administered before the picture block.

All 384 old items (192 pictures and 192 words) were presented randomly intermixed with 192 new items (96 pictures and 96 words). We chose a 2:1 ratio of old and new items in the test phase because only one half of the old items were attended during the encoding phase (targets) and the other half was not attended (distractors). The assignment of old and new items to one of the two test phases was counterbalanced across participants.

## 3. Results

Analyses of variance (ANOVAs) were used for analyzing performance in task switching (accuracy rates and reaction times in ms) and in the recognition test (proportion of correctly recognized pictures and words, i.e., hits). Effect sizes ($\eta_{p}^{2}$) represent partial eta squared.

### 3.1. Task switching

Table 1 presents mean accuracy rates and reaction times. Accuracy rates and reaction times were subjected to a 3 (stimulation) × 2 (transition) ANOVA. The expected switch costs emerged for both performance measures, but no effect of stimulation was evident. Participants answered more correctly on repeat (*M* = 0.945, *SE* = 0.004) than switch trials (*M* = 0.916, *SE* = 0.007), as indicated by a significant main effect of transition, *F* (1,57) = 31.60, *p* < 0.001, $\eta_{p}^{2}$ = 0.36. Neither the main effect of stimulation, nor the interaction were significant for accuracy, *F* (2,57) = 0.57, *p* = 0.567, $\eta_{p}^{2}$ = 0.02, and *F* (2,57) = 0.38, *p* = 0.684, $\eta_{p}^{2}$ = 0.01, respectively. Participants answered faster on repeat (*M* = 1060, *SE* = 23) than switch trials (*M* = 1624, *SE* = 34), as indicated by significant main effect of transition, *F* (1,57) = 443.94, *p* < 0.001, $\eta_{p}^{2}$ = 0.89. Neither the main effect of stimulation, nor the interaction were significant for reaction times, *F* (2,57) = 2.20, *p* = 0.120, $\eta_{p}^{2}$ = 0.07, and *F* (2,57) = 0.83, *p* = 0.440, $\eta_{p}^{2}$ = 0.03, respectively.

**Table 1.** Task switching performance. Means and standard errors (in parentheses) for accuracy as the proportion of correct answers and reaction times in ms.

Measure	Transition	Stimulation		
		Dorsal-Anodal	Sham	Ventral-Anodal
Accuracy	Repeat	0.945 (0.008)	0.951 (0.006)	0.940 (0.008)
	Switch	0.909 (0.012)	0.926 (0.010)	0.913 (0.013)
Reaction times	Repeat	1063 (40)	1127 (47)	990 (27)
	Switch	1673 (65)	1653 (57)	1546 (52)

### 3.2. Recognition test

**Figure 3.**
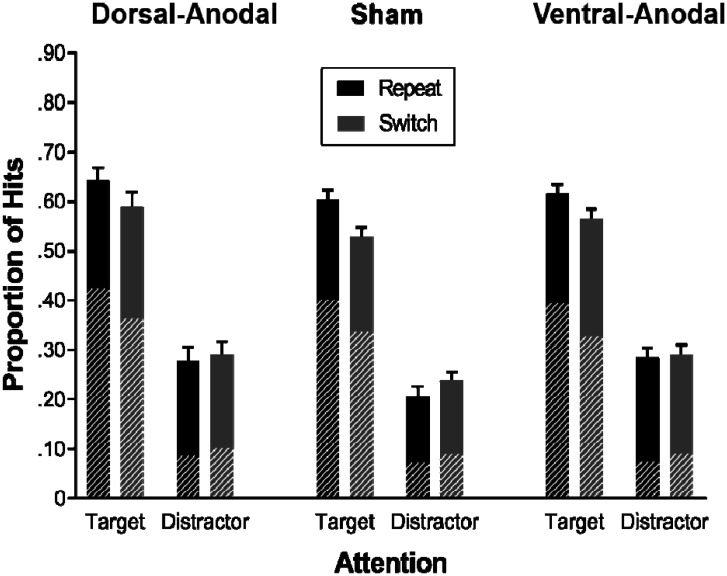
Memory performance. Mean proportion of correctly recognized old items (hits) as a function of stimulation (dorsal-anodal, sham, and ventral-anodal), attention (target vs. distractor), and transition (repeat vs. switch trial). The shaded areas reflect remember-responses, the solid areas reflect know-responses. Error bars represent standard errors.

Overall hit rates and false alarm rates were highest in the dorsal-anodal condition (hits: *M* = 0.450, *SE* = 0.027; false alarms: *M* = 0.201, *SE* = 0.037), followed by the ventral-anodal (hits: *M* = 0.438, *SE* = 0.026; false alarms: *M* = 0.183, *SE* = 0.029), and sham conditions (hits: *M* = 0.395, *SE* = 0.029; false alarms: *M* = 0.150, *SE* = 0.020). To test the effects of stimulation condition, attention and transition, we conducted three separate 3 × 2 × 2 ANOVAs for recognition performance, remember-responses, and know-responses (the results for remember-responses and know-responses are presented in Table 2). Descriptive statistics are depicted in Figure 3. Attended target stimuli (*M* = 0.591, *SE* = 0.013) were better remembered than unattended distractor stimuli (*M* = 0.265, *SE* = 0.012), as indicated by the main effect of attention, *F* (1,57) = 572.92, *p* < 0.001, $\eta_{p}^{2}$ = 0.91. Stimuli from repeat trials (*M* = 0.439, *SE* = 0.021) were better remembered than stimuli from switch trials (*M* = 0.417, *SE* = 0.018), as indicated by a main effect of transition, *F* (1,57) = 12.63, *p* < 0.001, $\eta_{p}^{2}$ = 0.18. The significant interaction between attention and transition, *F* (1,57) = 35.03, *p* < 0.001, $\eta_{p}^{2}$ = 0.38, represents the switch costs on memory selectivity; *better* target memory for repeat (*M* = 0.621, *SE* = 0.017) compared to switch trials (*M* = 0.561, *SE* = 0.019), but *worse* distractor memory for repeat (*M* = 0.257, *SE* = 0.017) compared to switch trials (*M* = 0.273, *SE* = 0.018). The main effect of stimulation was not significant, *F* (2,57) = 1.14, *p* = 0.326, $\eta_{p}^{2}$ = 0.04, nor were the interactions with attention, *F* (2,57) = 0.74, *p* = 0.480, $\eta_{p}^{2}$ = 0.03, transition, *F* (2,57) = 0.01, *p* = 0.985, $\eta_{p}^{2}$ < 0.01, or the three-way interaction, *F* (2,57) = 1.36, *p* = 0.264, $\eta_{p}^{2}$ = 0.05.

In order to assess the extent to which the data support the absence of stimulation effects we conducted a Bayesian analysis [27]. Using JASP (Version 0.13), we calculated a Bayesian ANOVA on recognition memory with the factors attention, transition, and stimulation. The Bayes Factors for all effects are presented in Table 3. The three-way interaction between attention, transition, and stimulation was the focus of the present study. Thus, we compared a model with the interaction to a model without the interaction. Including the three-way interaction in the model gives a Bayes Factor of 0.213, while excluding the three-way interaction gives a Bayes Factor of 4.702, suggesting that the data are 4.702 times more likely under the model without the three-way interaction than under the model that adds the interaction.

**Table 2.** Results of the recognition test for remember- and know-responses. Mean proportion of hits was analyzed by means of a 3 (stimulation: dorsal-anodal, sham, ventral-anodal) × 2 (attention: target vs. distractor) × 2 (transition: repeat vs. switch trial) analysis of variance (ANOVA). The same ANOVA was conducted separately for remember-responses and for know-responses. *η^2^_p_* indicates partial eta squared.

		Remember			Know		
Effects	*df*	*F*	*p*	*η^2^_p_*	*F*	*p*	*η^2^_p_*
Stimulation	2,57	0.30	0.74	0.01	1.52	0.23	0.05
Attention	1,57	414.15	<0.01	0.88	16.81	<0.01	0.23
Transition	1,57	29.10	<0.01	0.34	0.23	0.63	<0.01
Stimulation × Attention	2,57	0.19	0.83	0.01	0.99	0.38	0.03
Stimulation × Transition	2,57	0.02	0.98	<0.01	<0.01	>0.99	<0.01
Attention × Transition	1,57	52.15	<0.01	0.48	0.16	0.70	<0.01
Stimulation × Attention × Transition	2,57	0.04	0.96	<0.01	1.93	0.16	0.06

**Table 3.** Results of the Bayesian analysis of memory performance. Models containing the effect were compared to equivalent models stripped of the effect. Given the data, the Bayes Factor indicates the likelihood of the model including the effect to a model excluding the effect.

Effects	Bayes Factor
Stimulation	1.541964
Attention	1.321946e + 75
Transition	1.548470
Stimulation × Attention	0.098741
Stimulation × Transition	0.030167
Attention × Transition	428.716171
Stimulation × Attention × Transition	0.212671

## 4. Discussion

The present study aimed to disentangle the roles of two attentional systems for selective memory by applying oppositional tDCS over the superior parietal cortex (a substrate of selective attention) and inferior parietal cortex (a substrate of orienting) during task switching. The results revealed a robust task switching effect on attention and memory selectivity, replicating previous research [5],[6]. Compared to repeating a task, switching between two classification tasks led to longer reaction times and more errors, suggesting hampered attention control on switch trials. The subsequent recognition test revealed a corresponding task switching effect on memory: Worse target memory but better distractor memory for items presented on switch (vs. repeat) trials. That is, the difference between target memory and distractor memory was lower on switch than repeat trials, indicating that task switching reduces memory selectivity. As this effect was not modulated by the application of tDCS and we did not find any other tDCS effects, we conclude that the present tDCS protocol was not suitable to modulate task switching performance or memory performance.

This failure is somewhat surprising, as a large body of neuroimaging studies suggests that activation in ventral and dorsal parietal brain areas is associated with behavioral indicators of selective attention and subsequent memory effects [1],[2],[9],[12],[13]. Furthermore, several studies stimulated the parietal cortex by tDCS or a related method (i.e., transcranial magnetic stimulation) and were indeed successful in modulating attention and memory [10],[18],[28]–[30]. In fact, our tDCS protocol was identical to a previous study that found a memory benefit for the dorsal-anodal stimulation condition.

It is possible that differences in the study design and the materials are responsible for the lack of tDCS effects in the present study. To account for different study designs, we reanalyzed our data without the sham condition. However, the difference between the stimulation conditions was still not significant, suggesting that the sham condition did not mask any true stimulation effects. For practical reasons^1^ we had to vary stimulation between subjects. Varying stimulation within-subject may be critical as individual's cortical activity upon arrival for testing affect polarity effects [31]. For example, alertness and caffeine intake can interact with stimulation and even inverse the effects of anodal and cathodal stimulation [32]. It could be that individual differences in cortical excitability obliterated the effects of anodal and cathodal stimulation on the mean grouping level. Another difference lies in the duration of tDCS. In Jacobson et al.'s [10] study tDCS lasted for 10 min while in the present study tDCS lasted for a total of approximately 20 min. By starting tDCS 10 min before the critical task switching phase, we aimed to reduce inter-individual differences in cortical activity upon arrival and ensure that the stimulation is fully effective at the start of the critical task switching phase. This could present a critical methodological difference between studies.

Furthermore, materials and tasks differed considerably between studies. Jacobson et al. [10] used word lists and instructed participants to encode the words for a later recognition test. In our study, however, a task switching procedure served as the incidental encoding phase and participants had no knowledge about the upcoming recognition test. A recent meta-analysis suggests that the cranial-cranial electrode pair placement is not effective in modulating executive functions (as opposed to an extracranial-cranial montage) [33]. Thus, it could be that the difference in demands posed on executive functions may explain why the cranial-cranial oppositional tDCS protocol was not effective in the present study. The explicit memory task used in the Jacobson et al.'s study [10] poses less demands on executive functions than the task switching procedure used in the present study. Because tDCS interacts with the brain activity elicited by a specific task [34], and because participants reported that the task switching experiment was cognitively demanding, it could be that our task demands already engaged the attentional networks (and probably also executive functions) so intensively that tDCS had no further impact. This interpretation is in line with a recent meta-analysis that found small and non-significant tDCS effects on memory and it suggests that tDCS exerts its influence only under specific conditions [35].

## 5. Conclusion

A stimulation protocol that successfully modulated memory in a previous study [10], was not successful in the present study when applied during incidental encoding of study materials presented during task switching. The fact that tDCS exerts its effect in one paradigm but not in another suggests that tDCS effects are highly task-specific. This conclusion converges with the inconsistent literature on parietal and frontal tDCS effects on attention and memory [33]–[41]. More research is needed to better understand how tDCS interacts with task-specific brain activation effects. Studies that systematically vary stimulation protocols with identical tasks and studies that use identical stimulation protocols with different tasks may be fruitful in identifying the circumstances under which tDCS effects emerge.

## Footnotes

^1^Repeating the task switching procedure and the recognition test within-subject would make the second recognition test not so surprising anymore. Informing participants about any upcoming recognition tests would alter their attentional control during task switching because they could engage strategies for better encoding.
